# Management of cardiovascular disease using an mHealth tool: a randomized clinical trial

**DOI:** 10.1038/s41746-021-00535-z

**Published:** 2021-12-03

**Authors:** Si-Hyuck Kang, Hyunyoung Baek, Jihoon Cho, Seok Kim, Hee Hwang, Wonjae Lee, Jin Joo Park, Yeonyee E. Yoon, Chang-Hwan Yoon, Young-Seok Cho, Tae-Jin Youn, Goo-Yeong Cho, In-Ho Chae, Dong-Ju Choi, Sooyoung Yoo, Jung-Won Suh

**Affiliations:** 1 Division of Cardiology, Department of Internal Medicine, Seoul National University and Seoul National University Bundang Hospital, Seongnam, Republic of Korea; 2 Healthcare Information and Communication Technology Research Center, Office of eHealth Research and Businesses, Seoul National University Bundang Hospital, Seongnam, Republic of Korea; 3 Department of Pediatrics, Seoul National University Bundang Hospital, Seongnam, Republic of Korea; 4 Department of Cardiology, Incheon Sejong Hospital, Incheon, Republic of Korea

**Keywords:** Disease prevention, Vascular diseases

## Abstract

Atherosclerotic cardiovascular disease (ASCVD) is a leading cause of death and morbidity worldwide. This randomized controlled, single-center, open-label trial tested the impact of a mobile health (mHealth) service tool optimized for ASCVD patient care. Patients with clinical ASCVD were enrolled and randomly assigned to the intervention or control group. Participants in the intervention group were provided with a smartphone application named HEART4U, while a dedicated interface integrated into the electronic healthcare record system was provided to the treating physicians. A total of 666 patients with ASCVD were enrolled, with 333 patients in each group. The estimated baseline 10-year risk of cardiovascular disease was 9.5% and 10.8% in the intervention and control groups, respectively, as assessed by the pooled cohort risk equations. The primary study endpoint was the change in the estimated risk at six months. The estimated risk increased by 1.3% and 1.1%, respectively, which did not differ significantly (*P* = 0.821). None of the secondary study endpoints showed significant differences between the groups. A post-hoc subgroup analysis showed the benefit was greater if a participant in the intervention group accessed the application more frequently. The present study demonstrated no significant benefits associated with the use of the mHealth tool in terms of the predefined study endpoints in stable patients with ASCVD. However, it also suggested that motivating patients to use the mHealth tool more frequently may lead to greater clinical benefit. Better design with a positive user experience needs to be considered for developing future mHealth tools for ASCVD patient care.

**Trial Registration:** ClinicalTrials.gov NCT03392259

## Introduction

Atherosclerotic cardiovascular disease (ASCVD) is one of the leading causes of death and morbidity worldwide^1–3^. Optimal management—including lifestyle modifications, medications, and interventions—has been shown to improve long-term outcomes in patients with ASCVD^4,5^. However, optimal management targets have been difficult to achieve in the real world. Studies have shown that less than 50% of patients with ASCVD adhere to secondary prevention medications recommended by current guidelines^6^. Likewise, almost half of the patients were reported as not following the recommended levels of lifestyle modification^7,8^.

It is estimated that there were 3.2 billion smartphone users worldwide as of 2019^9^. The global smartphone penetration rate has crossed 40%, with higher rates in advanced countries (70.4% in Korea)^10^. Mobile phones have shown potential as tools to enhance patient awareness, promote physician-patient relationships, and ultimately improve risk factor management. While a variety of health-related applications have been developed, clinical validation has been scarce^11,12^. In addition, there have been inconsistent results regarding the benefits of using such applications for risk factor modification^13–16^.

A mobile health (mHealth) service tool optimized for cardiovascular disease patient care was developed to enhance self-engagement and patient-physician communication^17^. The tool included two end products, a smartphone application for patients and a dedicated electronic healthcare record (EHR) interface for their treating physicians. In this study, a randomized controlled trial was conducted to evaluate the impact of the mHealth tool in real-world practice.

## Results

### Baseline characteristics

**Fig. 1:**
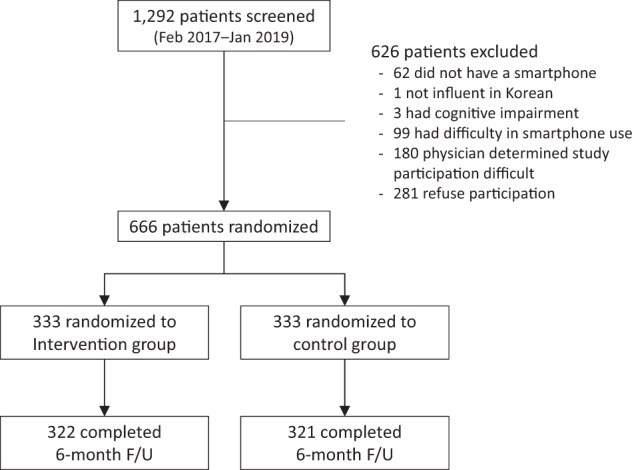
CONSORT study flow diagram. The diagram indicates the number of patients screened, enrolled, and randomized in the trial, and the study flow.

Between February 2018 and January 2019, a total of 1292 patients with ASCVD were screened, of whom 666 were enrolled in the trial (Fig. 1; inclusion and exclusion criteria, Supplementary Table 1). Patients were randomly assigned to the intervention or control group in a uniform 1:1 ratio. The control group was treated with guideline-based standard care including lifestyle counseling and medications^18–23^. The intervention group received mHealth-based interventions as well as standard care. The mean age was 58.3 years, and more than 80% of the patients were male (Table 1). One-fourth of the patients had a history of acute coronary syndrome. Most enrolled patients had been treated with coronary revascularization. The median time from the last coronary revascularization was 645 days (interquartile range = 174‒1,751 days).

**Table 1:** Baseline features of included patients.

	Total (*N* = 666)	Intervention group (*N* = 333)	Control group (*N* = 333)	*P* value
Age, years	58.3 ± 7.7	57.4 ± 7.7	59.2 ± 7.6	<0.01
Male sex	550 (82.6%)	279 (83.8%)	271 (81.4%)	0.47
Home BP measurements	232 (34.8%)	122 (36.6%)	110 (33.0%)	0.37
Antihypertensive medications	330 (49.5%)	165 (49.5%)	165 (49.5%)	>0.99
Diabetes	200 (30.0%)	92 (27.6%)	108 (32.4%)	0.15
History of myocardial infarction	111 (16.7%)	52 (15.6%)	59 (17.7%)	0.53
History of unstable angina	46 (6.9%)	25 (7.5%)	21 (6.3%)	0.35
History of coronary revascularization	648 (97.3%)	321 (96.4%)	327 (98.2%)	0.23
Total cholesterol (mg/dL)	141.8 ± 30.4 (666)	141.9 ± 28.3 (333)	141.8 ± 32.4 (333)	0.95
HDL cholesterol (mg/dL)	45.9 ± 11.4 (663)	46.5 ± 11.8 (332)	45.3 ± 10.9 (331)	0.11
LDL cholesterol (mg/dL)	81.4 ± 23.0 (660)	81.6 ± 22.4 (330)	81.1 ± 23.7 (330)	0.82
Systolic blood pressure (mmHg)	124.7 ± 16.4 (666)	124.6 ± 16.1 (333)	124.8 ± 16.7 (333)	0.89
Diastolic blood pressure (mmHg)	75.1 ± 10.8 (666)	75.4 ± 10.4 (333)	74.8 ± 11.2 (333)	0.49
Body mass index (kg/m2)	25.0 ± 3.2 (666)	25.5 ± 3.3 (333)	25.2 ± 3.1 (333)	0.28
Cigarette smoking	161 (24.2%)	66 (19.8%)	95 (28.8%)	<0.01
Predicted 10-year ASCVD risk (%)**	10.1 (5.5‒16.1)	9.5 (5.1‒15.3)	10.8 (5.8‒16.8)	0.01

^*^Mean ± SD (available number of subjects) for continuous variables, and *n* (%) for binary variables. **Median (interquartile ranges).

*BP* blood pressure, *HDL* high-density lipoprotein, *LDL* low-density lipoprotein, *ASCVD* atherosclerotic cardiovascular disease.

### Main study endpoints

Among the 666 study participants, 640 (96.1%) completed a 6-month visit. The primary study endpoint was the change in estimated 10-year risk of cardiovascular disease as assessed by the pooled cohort equations at six months (Supplementary Table 2). As shown in Table 1, the primary endpoint of estimated 10-year risks calculated by the pooled cohort equations was 9.5% (5.1‒15.3%) and 10.8% (5.8‒16.8%) at baseline for the intervention and control groups, respectively. The primary endpoint increased by 1.3% (4.1%) and 1.1% (5.1%), respectively, over six months after randomization (Table 2). The study hypothesis was not met with statistical significance (1-sided *P* = 0.821, 2-sided *P* = 0.358). The multivariable analysis adjusted for baseline risk factors showed no significant difference in the primary endpoint (*P* = 0.257). Any changes in conventional cardiovascular risks such as lipid profiles, blood pressure, body mass index, and smoking status did not differ significantly between the groups. Neither did the other secondary endpoints such as physical activity, depression, medication adherence, and patient activation.

**Table 2:** Primary and secondary study endpoints at baseline and at 6 months after randomization.

	Intervention group (*N* = 322)			Control group (*N* = 321)			*P* value
	Baseline	At 6 months	Difference	Baseline	At 6 months	Difference	
Predicted 10-year ASCVD risk (%)	9.3 (5.2‒14.8)	10.2 (6.3‒16.9)	1.3 ± 4.1	10.8 (5.7‒16.9)	11.2 (6.9‒17.9)	1.1 ± 5.1	0.633
Secondary endpoints: conventional cardiovascular risks							
Total cholesterol (mg/dL)	142.1 ± 28.4	140.5 ± 27.9	−1.7 ± 22.9	140.9 ± 32.1	139.7 ± 30.0	−1.2 ± 26.3	0.827
HDL cholesterol (mg/dL)	46.5 ± 11.9	47.7 ± 12.9	1.2 ± 10.0	45.3 ± 11.0	47.3 ± 11.5	2.1 ± 7.1	0.184
LDL cholesterol (mg/dL)	81.9 ± 22.5	79.9 ± 20.9	−2.0 ± 18.8	80.5 ± 23.3	79.6 ± 21.1	−0.9 ± 19.3	0.485
Systolic blood pressure (mmHg)	124.3 ± 16.0	126.2 ± 14.1	1.9 ± 16.8	125.0 ± 16.8	125.7 ± 16.8	0.7 ± 18.3	0.385
Diastolic blood pressure (mmHg)	75.2 ± 10.3	75.8 ± 10.3	0.5 ± 11.6	74.9 ± 11.3	75.3 ± 10.1	0.4 ± 11.6	0.915
Body mass index (kg/m^2^)	25.5 ± 3.3	26.6 ± 18.1	1.1 ± 17.9	25.2 ± 3.1	26.8 ± 19.6	1.6 ± 19.2	0.746
Cigarette smoking	63 (19.6%)	63 (19.6%)	0 (0%)	89 (27.7%)	88 (27.4%)	−1 (−0.2%)	0.499
Secondary endpoints: others							
Godin leisure-time exercise questionnaire	24.3 ± 19.7	21.5 ± 22.7	−2.8 ± 22.8	23.3 ± 22.8	19.7 ± 22.7	−3.6 ± 29.9	0.751
Patient health questionnaire-9	3.6 ± 4.1	2.0 ± 3.2	−1.6 ± 4.2	3.4 ± 3.5	2.0 ± 3.4	−1.4 ± 3.8	0.527
Beck depression inventory	7.7 ± 7.6	3.8 ± 5.5	−3.9 ± 7.2	7.2 ± 6.5	4.1 ± 5.8	−3.1 ± 6.0	0.108
Medication adherence rating scale	7.1 ± 1.5	7.1 ± 1.4	0.0 ± 1.8	7.4 ± 1.5	7.1 ± 1.	−0.3 ± 1.8	0.102
Patient activation measure-13	40.3 ± 5.5	40.3 ± 5.7	0.0 ± 7.4	39.7 ± 5.9	40.1 ± 5.6	0.4 ± 7.2	0.547

### Subgroup analysis, user experience, and application usage

Pre-planned subgroup analysis showed that the treatment effect had little variation across subgroups including age and sex (Supplementary Fig. 1). In addition, post-hoc subgroup analysis of any history of acute coronary syndrome or the time from the incident event to the enrollment did not show any significant interaction.

**Fig. 2:**
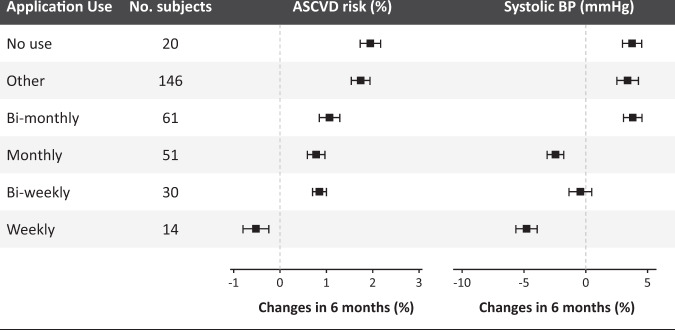
Subgroup analysis. Subgroup analysis results according to application use during the study period.

Participants in the intervention group generally expressed favorable to neutral satisfaction with the smartphone application (Supplementary Fig. 2). Log data showed that the participants accessed the smartphone application for a median of 11 days (interquartile range = 4‒32 days) during the study period. A greater clinical benefit was observed with more frequent access to the smartphone application in the intervention arm (Fig. 2). The increase in the primary endpoint at 6 months tended to be smaller with more frequent use (*P* for trend = 0.019). The trend was mainly driven by a greater reduction in systolic blood pressure (BP) (*P* for trend = 0.014).

### Factors affecting the intention to use

All the measurement variables had a Cronbach’s alpha value of 0.8 or higher and a composite reliability (omega) value of 0.8 or higher, indicating the reliability of the measurement indicators. As shown in the evaluation result of the measurement model, the composite reliability and convergent validity were also satisfied (Supplementary Table 3). The fitness of the hypothesized research model was suitable for all predefined criteria (Supplementary Table 4).

**Fig. 3:**
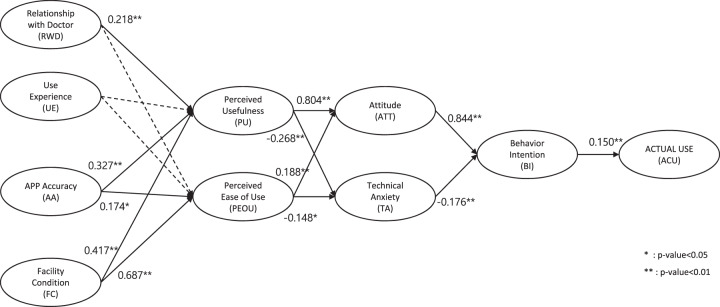
Factors affecting the application use. Proposed structural equation model and path coefficients for persistent application use.

The final model for factors affecting the use of the smartphone application resulting from path analysis is shown in Supplementary Table 5. All paths showed a significant relationship except for relationship with doctor to perceived usefulness, user experience to perceived usefulness, and user experience to perceived ease of use. Figure 3 shows the results of the adoption and intention to use the model of the study application.

## Discussion

The present study tested the clinical impact of mHealth-based individualized care in patients with ASCVD. Contrary to our expectations, no significant differences were found in terms of the pre-specified study endpoints. Exploratory analysis showed a trend toward a greater benefit with more frequent use of the smartphone application.

The World Health Organization defines mHealth as the use of mobile and wireless technologies to support the achievement of health objectives and provides positive prospects to transform health service delivery across the globe^24^. There is a wide spectrum of clinical aspects for which mHealth tools have been developed and are expected to play a role^25^. Recently, studies have reported the effectiveness of mHealth-based intervention in patients with cardiovascular disease. A study by Chow et al. showed that the use of lifestyle-focused text messaging resulted in a significant improvement in low-density lipoprotein (LDL)-C levels by 5 mg/dL in patients with coronary heart disease^13^. Blasco et al. reported promising results using a web-based telemonitoring system in patients who were discharged with acute coronary syndrome^14^. Several other studies that attempted to enhance medication adherence using mHealth tools in patients with ischemic heart disease showed conflicting results, some positive^26–28^, and others not^29,30^. Results from studies that sought to manage BP using mHealth tools have also been diverse^13,16,31,32^, although a meta-analysis suggested a significant reduction in systolic BP by 4.3 mmHg^15^.

Several factors may explain the absence of significant benefits of mHealth tools shown in this study. One factor is that the baseline risk of the study participants was lower than expected (10.1% at baseline vs. 13.5% expected). Modifiable risk factors were also close to the optimal levels: LDL cholesterol at 80 mg/dL; BP, 125/75 mmHg; body mass index at 25 kg/m;^2^ and ~20% were current smokers. Indeed, the risk profile was favorable compared to other studies in a secondary prevention setting^13,14,16,31,32^. For example, the guideline-recommended BP target is generally less than 130/80 mmHg for patients with stable cardiovascular disease^33,34^. Even in the SPRINT trial, where a standardized BP measurement was done, the mean systolic BP of the intensive treatment arm was 121 mmHg^35^. It is, therefore, unlikely that any mHealth intervention would make a significant difference in such populations.

The timing of intervention, however, may be of significance. Most participants were clinically stable patients with ASCVD. Approximately 60% were beyond one year after revascularization, whereas 80% were beyond three months. The benefits of mHealth interventions may be more apparent when implemented during or soon after admission. Although the post-hoc subgroup analysis of the present study does not support the idea, it is important to note that in this study, patients were enrolled only from outpatient clinics. Therefore, future studies need to address whether mHealth interventions have a greater impact in in-hospital admission settings. Secondary prevention including medical therapy and lifestyle modification is best adopted during admission or immediately after discharge, when patients are most motivated and when most treatment decisions are made.^36^^,^^37^

Despite these factors, it is disappointing that none of the secondary endpoints showed improvement, such as the Patient Activation Measure-13, which reflects patient knowledge, skills, and confidence in the management of chronic conditions^38^. The main purpose of the mHealth application was to enhance patients’ self-engagement^39^. Study participants in the intervention group accessed the application for a median of 11 days during the six-month study duration. Approximately half of the participants showed very low usage of the application, which indicates little to no exposure to the intervention.

A key takeaway from the study is that most patients do not enjoy being engaged with the mHealth tool. Previous studies have revealed challenges in mHealth use such as lack of knowledge on how to use mHealth tools, decreased sensory perception for patients, and increased data burden and workload for healthcare providers^40,41^. Availability of reliable devices and applications is another reported issue.41 This study exemplifies a similar difficulty in engaging patients with mHealth tools and changing their behaviors and health outcomes.

Another important insight from this study is that a greater benefit can be expected if a user accesses the smartphone application more frequently. According to one survey, after downloading health-related applications, almost half of the users stop using them^42^. The study showed that a quarter of participants in the intervention arm used the application for three days or less. Many factors have been reported to affect a user’s decision to continue using health-related applications^42,43^. Applications with better user experience, physicians’ commitment, and higher integration into healthcare systems could help maximize the utility of the mHealth approach.

We found that several factors, including user-friendly features, affect application usage. In the research model proposed in our study, it was found that application accuracy and facilitating conditions had a significant effect on perceived usefulness and perceived ease of use. These two factors were shown to positively impact attitude and reduce technical anxiety, which in turn resulted in persistent application use. Previous studies have also shown that these were among the most important factors that affect the acceptance of mHealth technology^44,45^.

Previous studies have also highlighted that adherence to medications is often poor in this patient group and hypothesized that smartphone technology, which is more ubiquitous, may help medication adherence; this may ultimately lead to an improvement in clinical outcomes. However, many factors other than forgetfulness are also at play in medication adherence^46^. Admittedly, smartphone applications alone can hardly address issues such as socioeconomic factors and healthcare system-related issues.

Although the present study reveals important findings, it has several limitations, the first of which is selection bias. Patient recruitment was conducted in a tertiary referral center, and 52% of the screened patients were enrolled in the trial. Studies have shown that higher socioeconomic levels are associated with a greater likelihood of clinical trial enrollment^47^. Similarly, eligibility criteria such as possession of a smartphone and familiarity with its use would have affected the study enrollment by privileging subjects with higher socioeconomic status such as income and education. The study results therefore cannot be generalized to other populations that were not represented in this study. Second, the baseline risk profile was lower than expected, as described above. Third, the pooled cohort equations, which were used as the primary endpoint assessment tool in this study, were originally developed for primary prevention settings. Lastly, the study period was only six months, and we cannot exclude the possibility that novel findings could emerge over a longer period.

In conclusion, an mHealth-based intervention did not show significant improvements in conventional cardiovascular risk factors in this study. However, an mHealth-based approach integrated into a local EHR was shown to be feasible and generally satisfactory to participants. Users who accessed the smartphone application more frequently were shown to have a higher benefit in terms of the study endpoint. This study suggests the importance of enhanced user experience and efforts to promote continued use of mHealth tools.

## Methods

### Study design

This study was a prospective randomized, single-center, open-label trial evaluating the efficacy of mHealth-based care compared with the standard care of patients with established ASCVD. The study was performed at Seoul National University Bundang Hospital, a tertiary referral center in Korea. The study conformed to the principles of the Declaration of Helsinki (revised version 2013). The study protocol was approved by the institutional review board of Seoul National University Bundang Hospital in November 2017 (B-1712-438-307). The study protocol is registered at ClinicalTrials.gov (ID: NCT03392259).

### Study population

Patients were eligible for participation if they were between 30 and 79 years of age, had clinical ASCVD, had a mobile smartphone on which the study application could be installed, and were sufficiently fluent in the Korean language to provide informed consent and understand the operation of smartphone applications (Supplementary Table 1). Clinical ASCVD was defined as (1) a history of acute myocardial infarction, unstable angina, or angina pectoris, (2) having received arterial revascularization therapy including coronary intervention, coronary artery bypass surgery, and peripheral artery angioplasty or surgery, (3) a history of stroke, or (4) peripheral arterial disease presumed to be of atherosclerotic origin^48–50^. The exclusion criteria were (1) cardiovascular disease of non-atherosclerotic origin, (2) cognitive impairment such as dementia, delirium, and amnestic disorder, (3) difficulty in using smartphone applications, and (4) other reasons that researchers determined would make participating in the study difficult.

All study participants provided informed consent before randomization. Patients were recruited by referral and enrolled by the investigators. Randomization was done using a web-based computerized program that was accessible to study personnel with a designated username and password. Intervention was initiated immediately after randomization. The treating physicians and study participants were not blinded to the assigned arm.

### Study process

The purpose, development, and details of the mHealth tool have been described previously^17^. The smartphone application, named HEART4U, works on both Android and iOS operating systems. It promotes patient self-engagement by providing relevant information and user-device interactions. As shown in Supplementary Figure 3, the six main menu items were (1) Goal, (2) Results, (3) Diary, (4) Survey, (5) Education, and (6) 1:1 Q&A (one-on-one question and answer feature). The Goal tab suggested individual targets for BP, physical activity, and body weight in comparison with current values. Users were prompted to measure their own BP levels regularly. Measured BP values could be entered into the application manually or automatically delivered from a Bluetooth-enabled home BP device. Detailed information on prescribed medications was also provided. An alarm service was available to remind users to take their medication. Laboratory results and estimated 10-year cardiovascular event risk assessed by pooled cohort equations were shown on the Results tab^48^. Users were encouraged to input their symptom changes and respond to self-administered questionnaires prior to an outpatient visit on the Diary tab. Video clips for patient education were also available on the Education tab. The 1:1 Q&A tab was for urgent inquiries. Messages, from which users could opt out, were sent every week delivering medical information on ASCVD and promoting positive lifestyle behaviors.

A dedicated user interface for the treating physicians was available on the EHR system (Supplementary Figure 4). Data collected via the application were summarized, including BP, drug adherence, and patients’ symptoms as well as key laboratory results. Physicians were able to adjust individual targets for BP, blood glucose levels, weight, physical activity, and smoking.

### Outcome measures

The primary study endpoint was the change in the estimated 10-year risk of cardiovascular disease at six months. The pooled cohort equations were developed to estimate the 10-year risk of developing a first ASCVD event among adults aged 40–79 years^48–50^. Although not intended for use in secondary prevention settings, it includes the most important modifiable risk factors such as blood cholesterol, BP, diabetes, and smoking status. In addition, its performance has been validated in secondary prevention settings^51–53^.

Secondary endpoints included systolic and diastolic BP (at three and six months), lipid profiles including total cholesterol, LDL cholesterol, and high-density lipoprotein (HDL) cholesterol, body mass index (kg/m^2^), smoking status, physical activity^54^, depression^55^, medication adherence^56^, patient activation^38^, and overall satisfaction with the application.

### Sample size calculation and statistical analysis

The trial was powered for assessing the superiority of mHealth-based care to standard care with respect to the primary endpoint at six months. A baseline 10-year ASCVD risk was assumed to be 13.5% (9.9%) based on the 1,423 patients who received coronary interventions at the study institution during the preceding year, 2016. The study hypothesis was that the estimated risk would reduce by 2.0% in the intervention arm and by 0.0% in the control arm at six months. With a sample size of 333 patients in each group, an attrition rate of 10%, and a one-sided significance level of 0.05, the study would provide a statistical power of 80% to detect the difference.

Statistical analysis was performed on an intention-to-treat basis. The primary endpoint was compared using an independent Student’s *t* test. Secondary endpoints were compared using Student’s *t* tests for continuous variables (BP, cholesterol, body mass index, physical activity, depression severity, medication adherence, overall satisfaction) or χ^2^ tests for categorical variables (smoking status). The mean levels (or proportions), 95% confidence intervals, and two-sided *P* values were provided for baseline characteristics and secondary study endpoints. Owing to significant imbalance in some baseline risk profiles, multivariable analysis was conducted for the primary endpoint adjusting the age, sex, and smoking status. Post-hoc subgroup analysis was performed according to age, sex, presence of home BP monitoring device, antihypertensive medication, diabetes, physical activity, and depression scales. The treatment effect was compared based on the days of application use derived from log data, which was categorized as weekly, biweekly, monthly, bimonthly, other, and no use. “Weekly” indicated at least one use every week, whereas “other” indicated less than one use in two months. “No use” indicated zero or one use during the overall study period. The R programming version 3.3.1 (R Foundation for Statistical Computing, Vienna, Austria) was used for sample size calculation and statistical analysis.

### Persistent use after completion of the study

A post-hoc analysis was done to investigate the factors affecting the intention to use the smartphone application. Participants in the intervention arm were asked to respond to a user experience survey which consisted of 25 questions covering the following nine domains: perceived usefulness (PU), perceived ease of use (PEOU), relationship with doctor (RWD), user experience (UE), application accuracy (AA), facilitating condition (FC), attitude (ATT), technical anxiety (TA), and behavior intention (BI). Responses were rated on a five-point Likert-type scale. The survey was based on previous theoretical models based on patients’ acceptance of mHealth^57,58^.

The factors affecting the intention to use the application were analyzed using structural equation modeling (SEM)^59^. The research model hypothesized in this study is shown in Supplementary Fig. 5.

Of the 332 participants in the intervention arm who completed a six-month visit, 20 who did not use the app during the study period and 12 who did not respond to more than 50% of the questionnaire were excluded; hence the modeling analyzed 290 participants (Supplementary Fig. 6). An R package lavaan (version 0.6–5) was used for the analysis^60^. The reliability and validity of the measurement model were assessed. For the validity, convergent validity and discriminant validity were assessed. Composite reliability (≥0.70) and indicator reliability (standardized loadings ≥ 0.70) were used as reliability evaluation indicators. Convergent validity was evaluated based on the significance of the indicator loadings (z-value >2) and the average variance extracted (≥0.50). Discriminant validity was evaluated based on the Fornell-Larcker criterion (square root of the average variance extracted of each latent variable is larger than its correlations with the other latent variables). The structural model was assessed through path analysis and model fit assessment. The path analysis was evaluated as a significant path for *P* < 0.05 for the path coefficient. For model fit assessment, the chi-square distribution (*P* < 0.05), standardized root-mean-square residual (<0.1), root-mean-square error of approximation (<0.1), comparative fit index (>0.9), normed fit index (>0.9), and the Tucker-Lewis index (>0.9) were used.

### Reporting summary

Further information on research design is available in the Nature Research Reporting Summary linked to this article.

## Supplementary information

Supplementary Information

Reporting Summary

## Data Availability

The data that support the findings of this study are available from the corresponding author upon reasonable request.
