# Supplementary material for: Management of cardiovascular disease using an mHealth tool: a randomized clinical trial
Source: NPJ Digit Med. 2021 Dec 3;4:165. doi: 10.1038/s41746-021-00535-z (PMC8642390; doi:10.1038/s41746-021-00535-z)
Supplement: Supplementary file 1 — Supplementary Information [file 41746_2021_535_MOESM1_ESM.pdf]

**Supplementary Figure 1.** Pre-planned subgroup analysis

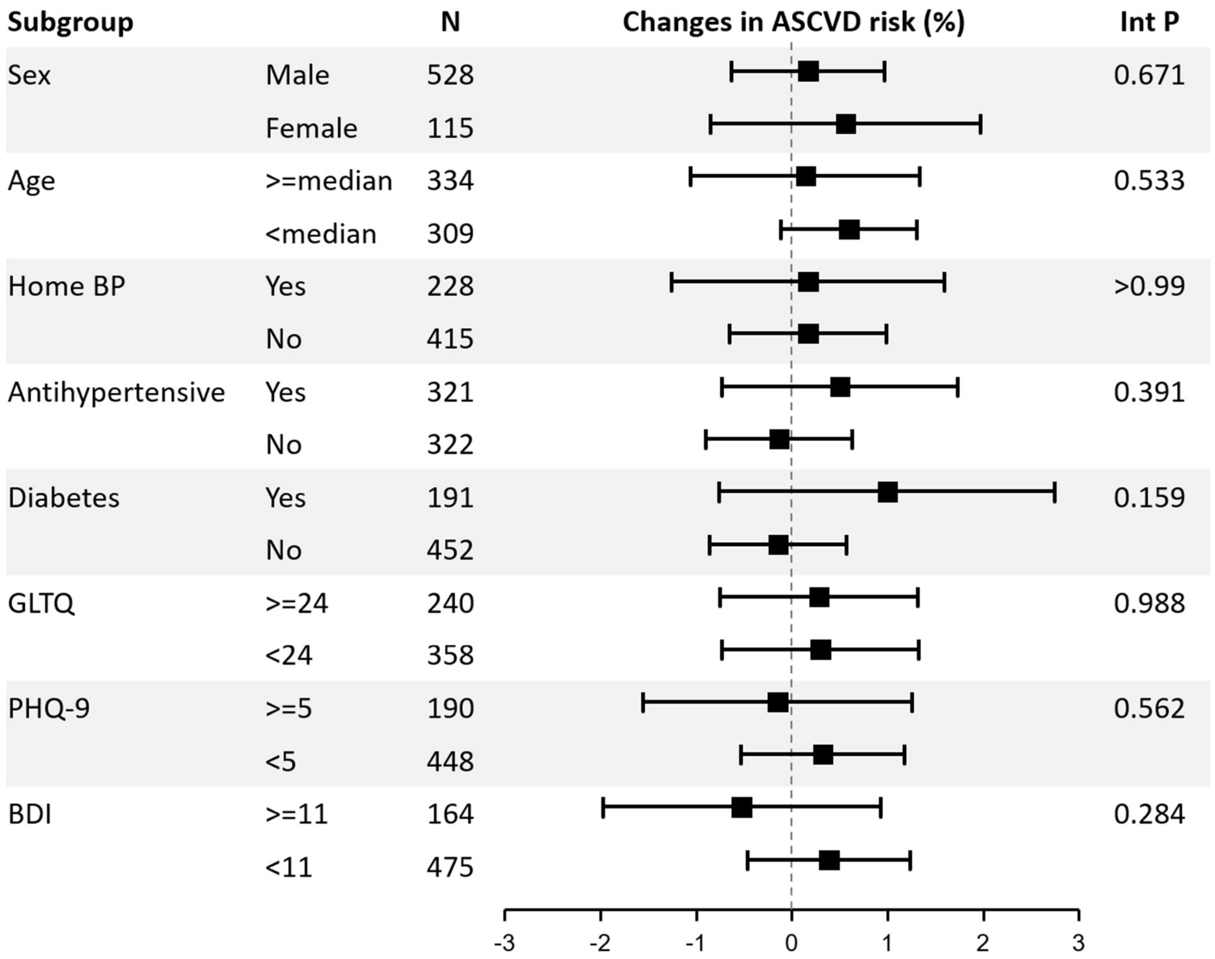


Abbreviations: BP, blood pressure; GLTQ, Godin Leisure-Time Exercise Questionnaire; PHQ-9, Patient Health Questionnaire-9; BDI, Beck depression inventory

**Supplementary Figure 2.** User experience survey results

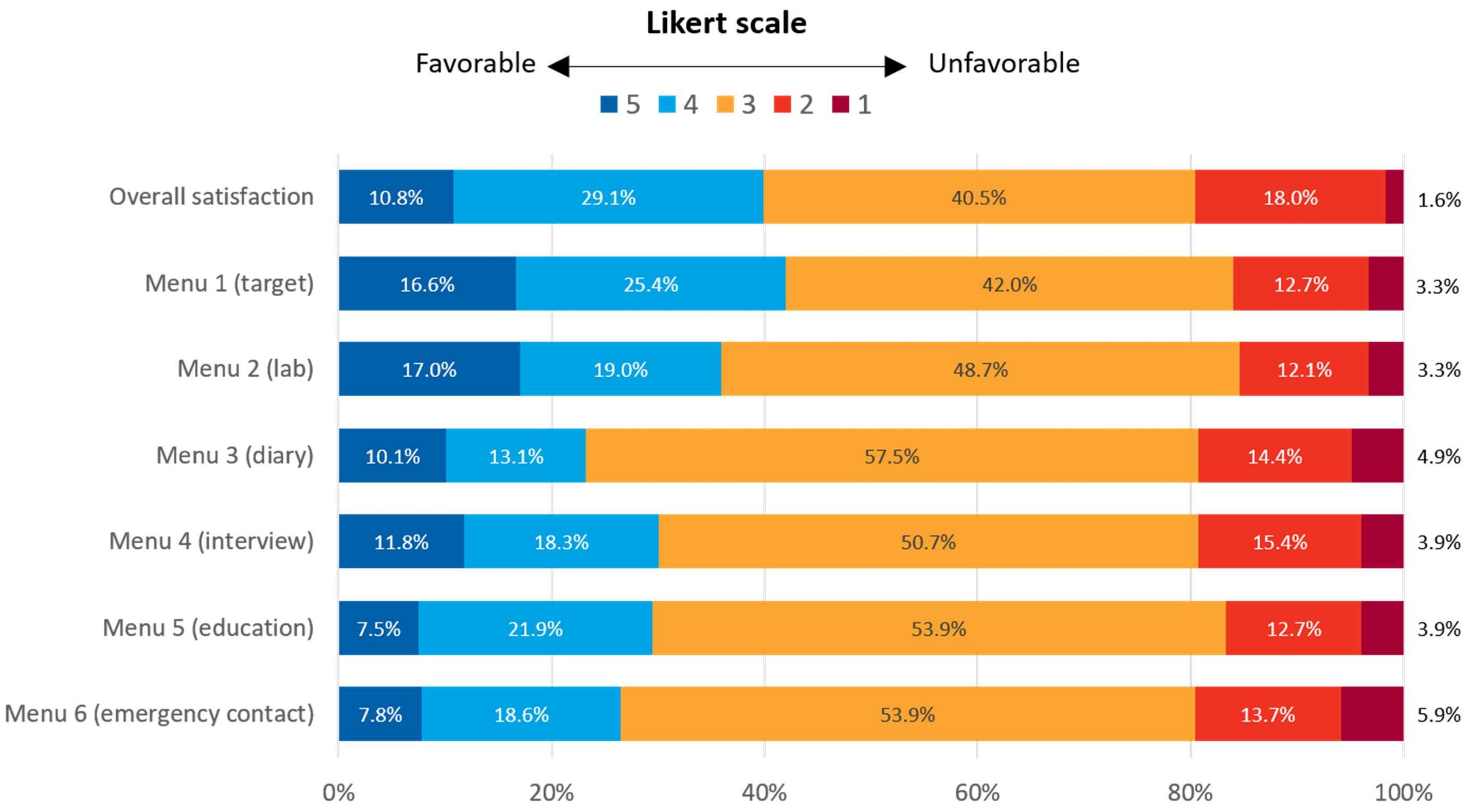

**Supplementary Figure 3.** User interface for patients

(A) Main screen, (B) target goals, (C) blood pressure input, (D) prescribed medications, (E) estimated 10-year cardiovascular event risk, (F) symptom diary, (G) self-administered questionnaires, (H) education contents, and (I) interface for treating physicians

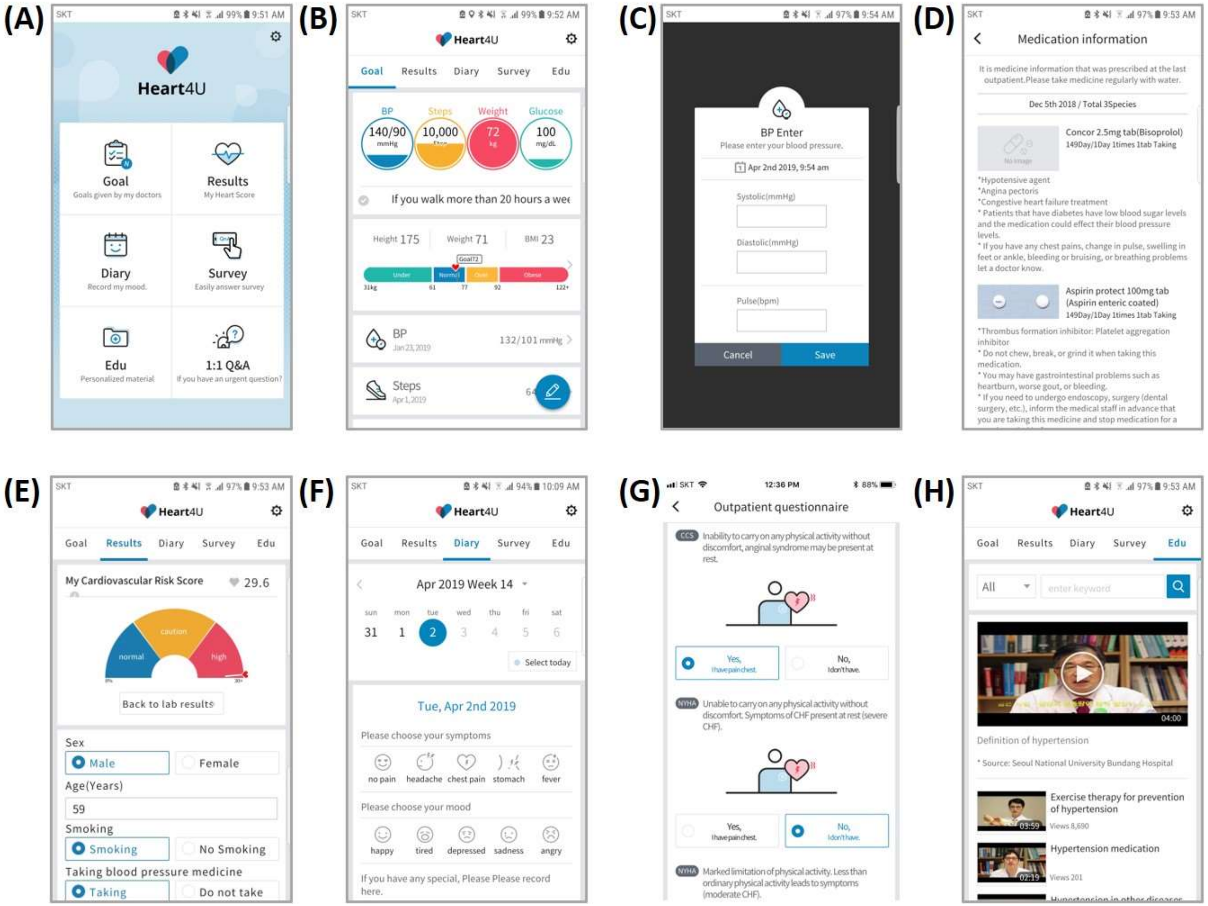

**Supplementary Figure 4.** User interface for patients physicians integrated into the electronic health record system

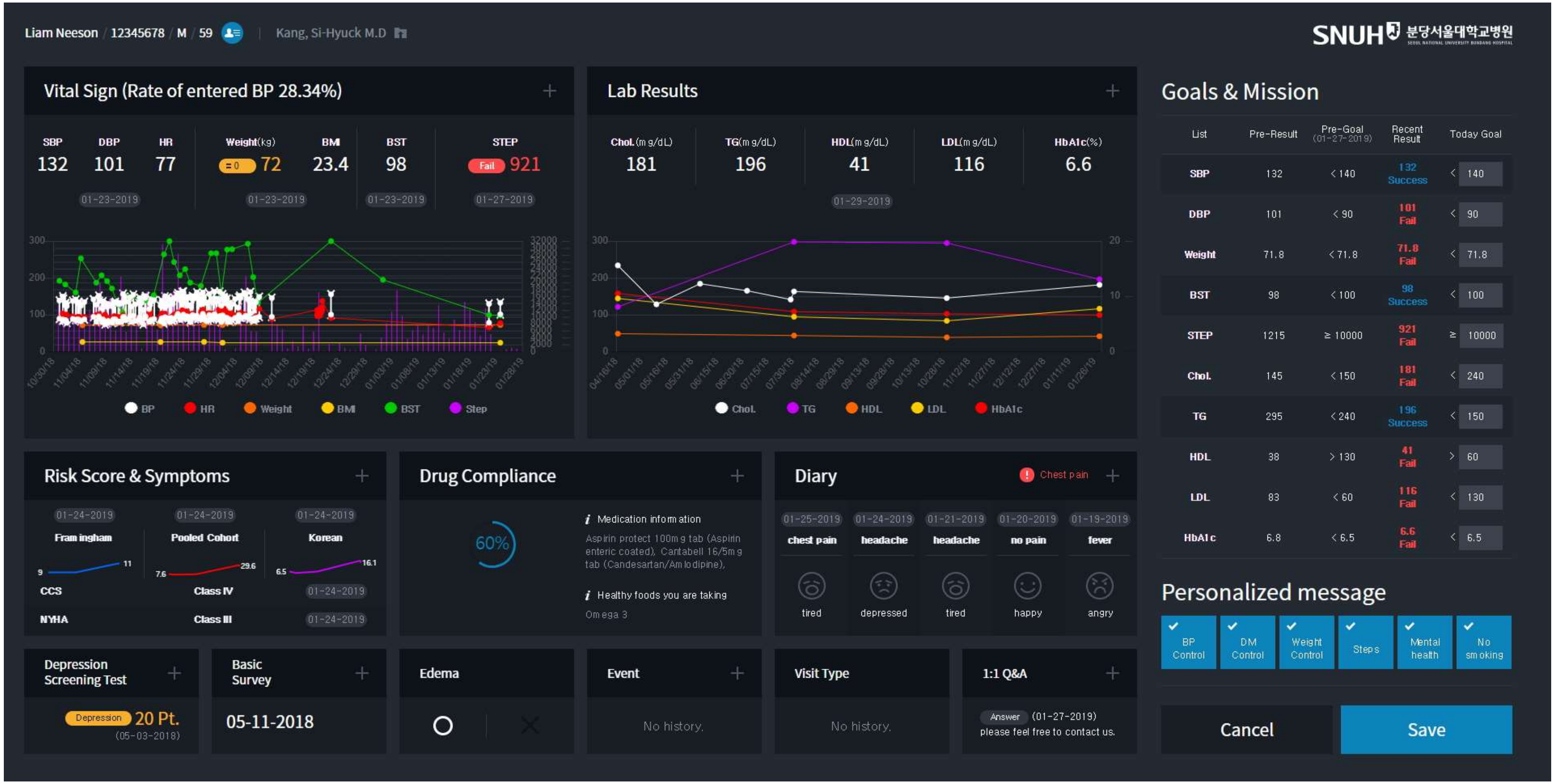

**Supplementary Figure 5.** Research model for patients' acceptance of the mHealth app

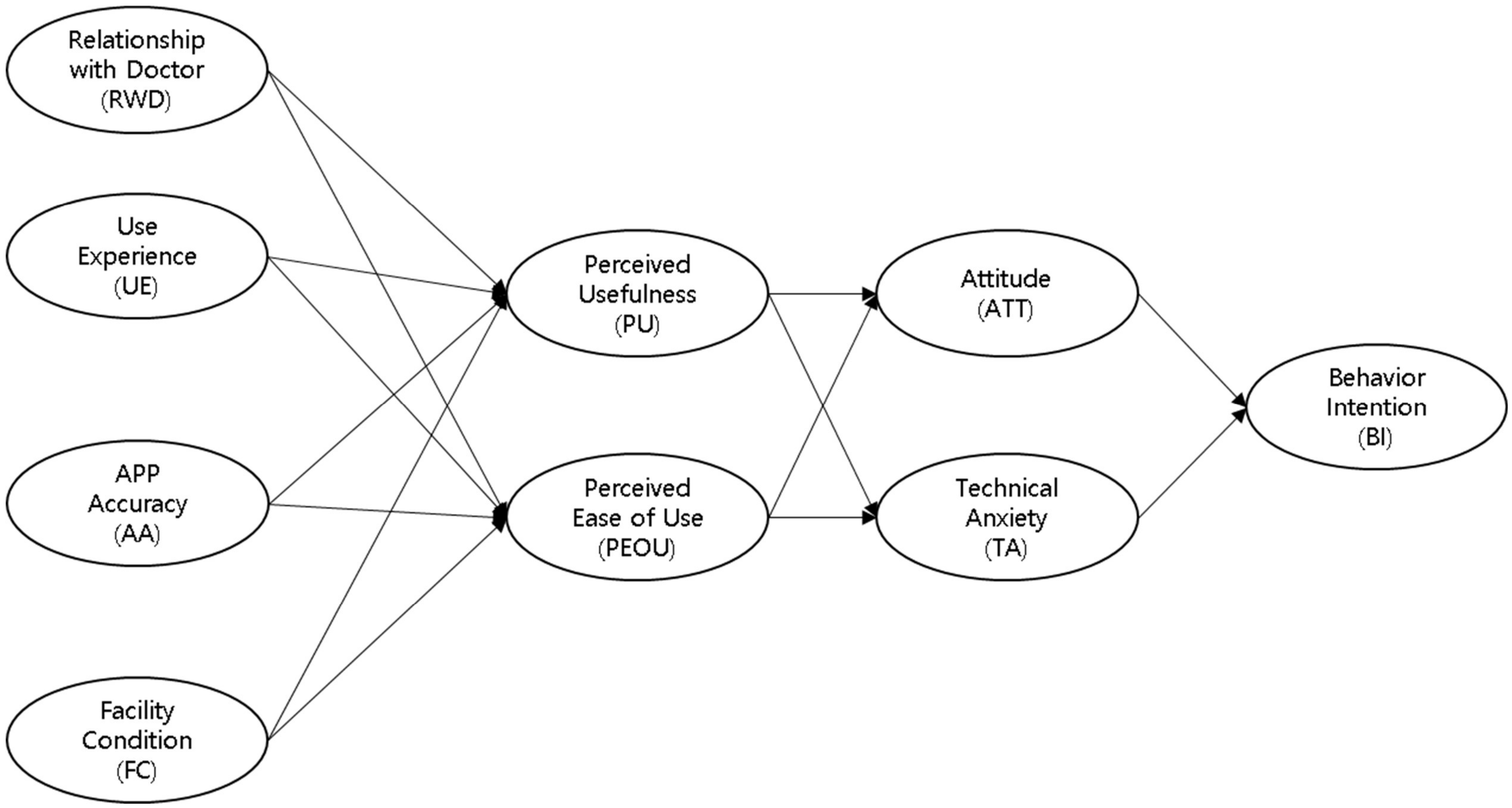

**Supplementary Figure 6.** Participants analyzed for structural equation model

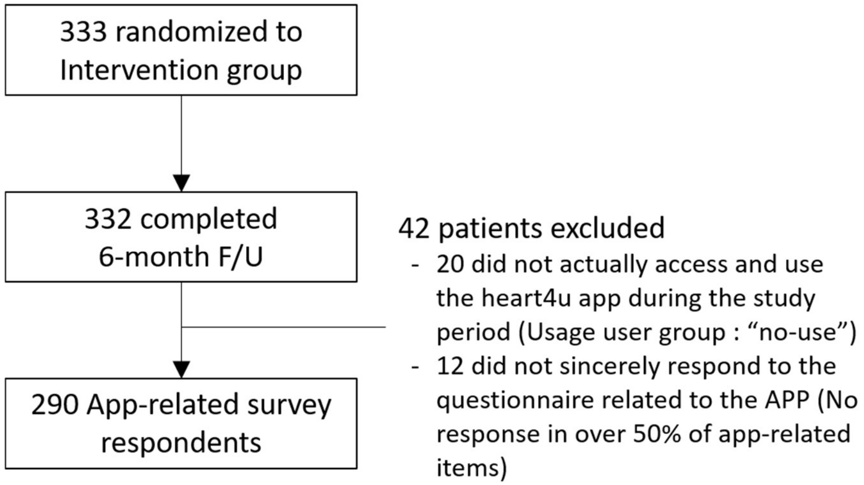

**Supplementary Table 1.** Inclusion and exclusion criteria

| **Inclusion criteria** |
|---|
| 1. Age between 30 and 79 years |
| 2. Clinical atherosclerotic cardiovascular disease defined by any of the followings: |
| 1) History of acute myocardial infarction, unstable angina, or angina pectoris |
| 2) History of arterial revascularization therapy including coronary intervention, coronary artery bypass surgery, and peripheral artery angioplasty or surgery |
| 3) history or stroke |
| 4) peripheral arterial disease presumed to be of atherosclerotic origin. |
| 3. Possession of a mobile smartphone where the study app can be installed, |
| 4. Sufficient Korean language skills to provided informed consent and understand the operation of smartphone apps. |

| **Exclusion criteria** |
|---|
| 1. Cardiovascular disease of non-atherosclerotic origin |
| 2. Cognitive impairment such as dementia, delirium, and amnestic disorder |
| 3. Difficulty in using smartphone apps |
| 4. Other reasons for which researchers determine participating in the study difficult. |

**Supplementary Table 2.** Study endpoints

| Endpoints | Measurement | Time frame |
|---|---|---|
| Primary endpoint | | |
| Change of predicted 10-year cardiovascular risk | Pooled cohort equations | 6 (±2) months |
| | | |
| Secondary endpoints | | |
| Systolic and diastolic blood pressure | Automated blood pressure measurements (mmHg) | 3 (±1) months<br>6 (±2) months |
| Lipid profiles | Total cholesterol (mg/dL)<br>Low-density lipoprotein cholesterol (mg/dL)<br>High-density lipoprotein cholesterol (mg/dL) | 6 (±2) months |
| Body mass index | Weight divided by (height)^2 (kg/$m^2$) | 6 (±2) months |
| Smoking status, | Proportion of active smoking (%) | 6 (±2) months |
| Physical activity | Godin Leisure-Time Exercise Questionnaire, total score (0–7) | 6 (±2) months |
| Depression severity | Patient Health Questionanaire-9 (PHQ-9), total score 27 (0–27) | 6 (±2) months |
| Medication adherence | Medication Adherence Rating Scale (MARS), total score 10 (0–10) | 6 (±2) months |
| Overall satisfaction survey over the app | Patient Activation Measure-13, total score 39 (0–39) | 6 (±2) months |

**Supplementary Table 3**. Evaluation result of the measurement model (reliability and convergent validity)

| lhs | op | rhs | est.std | est | se | z | pvalue | ci.lower | ci.upper | alpha | omega | avevar |
|---|---|---|---|---|---|---|---|---|---|---|---|---|
| RWD | =~ | APP_B_9_1 | 0.978 | 1.013 | 0.016 | 62.548 | 0 | 0.948 | 1.009 | 0.915 | 0.92 | 0.851 |
| RWD | =~ | APP_B_9_2 | 0.863 | 0.889 | 0.021 | 41.963 | 0 | 0.822 | 0.903 | | | |
| UE | =~ | APP_B_5_1 | 0.94 | 1.01 | 0.020 | 47.11 | 0 | 0.901 | 0.979 | 0.893 | 0.895 | 0.811 |
| UE | =~ | APP_B_5_2 | 0.858 | 0.904 | 0.023 | 36.798 | 0 | 0.812 | 0.904 | | | |
| AA | =~ | APP_B_13_1 | 0.917 | 0.822 | 0.017 | 52.739 | 0 | 0.882 | 0.951 | 0.890 | 0.890 | 0.802 |
| AA | =~ | APP_B_13_2 | 0.875 | 0.803 | 0.020 | 44.238 | 0 | 0.836 | 0.913 | | | |
| FC | =~ | APP_B_6_1 | 0.866 | 0.963 | 0.020 | 44.443 | 0 | 0.828 | 0.904 | 0.822 | 0.841 | 0.648 |
| FC | =~ | APP_B_6_2 | 0.916 | 0.935 | 0.016 | 57.016 | 0 | 0.884 | 0.947 | | | |
| FC | =~ | APP_B_6_3 | 0.580 | 0.593 | 0.043 | 13.539 | 0 | 0.496 | 0.664 | | | |
| PU | =~ | APP_B_1_1 | 0.938 | 0.885 | 0.010 | 91.934 | 0 | 0.918 | 0.958 | 0.939 | 0.940 | 0.839 |
| PU | =~ | APP_B_1_2 | 0.922 | 0.873 | 0.012 | 79.346 | 0 | 0.899 | 0.944 | | | |
| PU | =~ | APP_B_1_3 | 0.886 | 0.822 | 0.015 | 58.838 | 0 | 0.856 | 0.915 | | | |
| PEOU | =~ | APP_B_2_1 | 0.932 | 0.948 | 0.009 | 100.666 | 0 | 0.914 | 0.950 | 0.965 | 0.965 | 0.902 |
| PEOU | =~ | APP_B_2_2 | 0.969 | 1.000 | 0.006 | 160.989 | 0 | 0.957 | 0.981 | | | |
| PEOU | =~ | APP_B_2_3 | 0.947 | 0.956 | 0.008 | 121.271 | 0 | 0.932 | 0.963 | | | |
| ATT | =~ | APP_B_3_1 | 0.903 | 0.926 | 0.013 | 67.437 | 0 | 0.877 | 0.930 | 0.928 | 0.929 | 0.813 |
| ATT | =~ | APP_B_3_2 | 0.928 | 0.952 | 0.011 | 83.202 | 0 | 0.906 | 0.949 | | | |
| ATT | =~ | APP_B_3_3 | 0.873 | 0.893 | 0.016 | 53.381 | 0 | 0.841 | 0.905 | | | |
| TA | =~ | APP_B_7_1 | 0.890 | 0.898 | 0.018 | 48.086 | 0 | 0.854 | 0.926 | 0.887 | 0.894 | 0.740 |
| TA | =~ | APP_B_7_2 | 0.950 | 1.020 | 0.016 | 61.252 | 0 | 0.920 | 0.981 | | | |
| TA | =~ | APP_B_7_3 | 0.728 | 0.778 | 0.031 | 23.51 | 0 | 0.667 | 0.788 | | | |
| BI | =~ | APP_B_14_1 | 0.988 | 1.049 | 0.006 | 158.222 | 0 | 0.976 | 1.000 | 0.973 | 0.973 | 0.947 |
| BI | =~ | APP_B_14_2 | 0.958 | 1.021 | 0.008 | 124.701 | 0 | 0.943 | 0.973 | | | |

**Supplementary Table 4.** Evaluation results of the model fit

| Model fit Criteria | Proposed model value |
|---|---|
| Chi-square(X2) $p < 0.05$ | <0.001 |
| Standardized RMR (SRMR) < 0.1 | 0.066 |
| Root Mean Square Error of Approximation(RMSEA) < 0.1 | 0.088 |
| Comparative Fit Index (CFI) > 0.9 | 0.935 |
| Normed Fix Index (NFI) > 0.9 | 0.907 |
| Tucker-Lewis Index (TLI) > 0.9 | 0.922 |

**Supplementary Table 5.** Factors affecting the intention to use of the app evaluated using the structural equation

| | RWD | UE | AA | FC | PU | PEOU | ATT | TA | BI | SQRT_AVE |
|---|---|---|---|---|---|---|---|---|---|---|
| **RWD** | 1 | 0.559 | 0.677 | 0.617 | 0.635 | 0.552 | 0.698 | -0.443 | 0.707 | 0.923 |
| **UE** | 0.559 | 1 | 0.592 | 0.730 | 0.504 | 0.611 | 0.604 | -0.166 | 0.532 | 0.901 |
| **AA** | 0.677 | 0.592 | 1 | 0.630 | 0.659 | 0.603 | 0.728 | -0.278 | 0.797 | 0.895 |
| **FC** | 0.617 | 0.730 | 0.630 | 1 | 0.671 | 0.787 | 0.732 | -0.276 | 0.594 | 0.805 |
| **PU** | 0.635 | 0.504 | 0.659 | 0.671 | 1 | 0.713 | 0.875 | -0.347 | 0.716 | 0.916 |
| **PEOU** | 0.552 | 0.611 | 0.603 | 0.787 | 0.713 | 1 | 0.712 | -0.343 | 0.610 | 0.950 |
| **ATT** | 0.698 | 0.604 | 0.728 | 0.732 | 0.875 | 0.712 | 1 | -0.460 | 0.801 | 0.902 |
| **TA** | -0.443 | -0.166 | -0.278 | -0.276 | -0.347 | -0.343 | -0.460 | 1 | -0.469 | 0.860 |
| **BI** | 0.707 | 0.532 | 0.797 | 0.594 | 0.716 | 0.610 | 0.801 | -0.469 | 1 | 0.973 |
